# Advances in antibody discovery from human BCR repertoires

**DOI:** 10.3389/fbinf.2022.1044975

**Published:** 2022-10-20

**Authors:** Zichang Xu, Hendra S. Ismanto, Hao Zhou, Dianita S. Saputri, Fuminori Sugihara, Daron M. Standley

**Affiliations:** ^1^ Department of Genome Informatics, Research Institute for Microbial Diseases, Osaka University, Suita, Japan; ^2^ Core Instrumentation Facility, Immunology Frontier Research Center, Osaka University, Suita, Japan; ^3^ Department Systems Immunology, Immunology Frontier Research Center, Osaka University, Suita, Japan

**Keywords:** antibody, antigen, B cell sorting, B cell receptor (BCR), next generation sequencing (NGS), repertoire analysis, epitope, machine learning

## Abstract

Antibodies make up an important and growing class of compounds used for the diagnosis or treatment of disease. While traditional antibody discovery utilized immunization of animals to generate lead compounds, technological innovations have made it possible to search for antibodies targeting a given antigen within the repertoires of B cells in humans. Here we group these innovations into four broad categories: cell sorting allows the collection of cells enriched in specificity to one or more antigens; BCR sequencing can be performed on bulk mRNA, genomic DNA or on paired (heavy-light) mRNA; BCR repertoire analysis generally involves clustering BCRs into specificity groups or more in-depth modeling of antibody-antigen interactions, such as antibody-specific epitope predictions; validation of antibody-antigen interactions requires expression of antibodies, followed by antigen binding assays or epitope mapping. Together with innovations in Deep learning these technologies will contribute to the future discovery of diagnostic and therapeutic antibodies directly from humans.

## 1 Introduction

**FIGURE 1:**
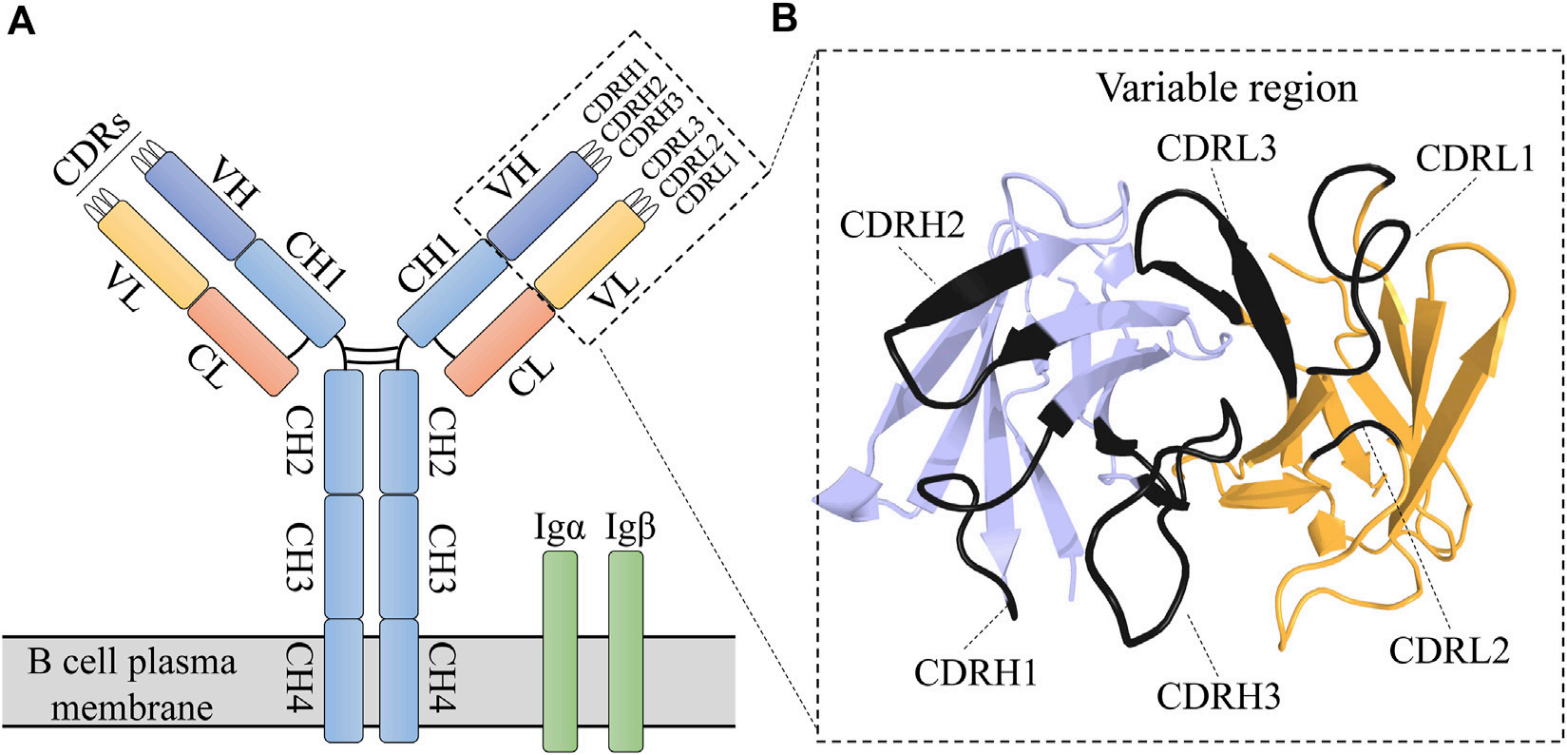
BCR structure. **(A)** Schematic representation of BCR structure. A BCR is composed of an immunoglobulin (antibody) molecule and a heterodimer (Igα/Igβ) that contain transmembrane and signal transduction regions. **(B)** The immunoglobulin variable region is composed of heavy (blue) and light (orange) chains (PDB entry: 7jmpHL). The six CDRs are represented by darker shades.

Antibodies, which are the extracellular portion of B cell receptors (BCRs), play a critical role in adaptive immune responses. An antibody consists of two chains, heavy and light, each of which is composed of a constant and a variable region (Figure 1). The six complementarity determining regions (CDR) of the variable region are responsible for binding a specific antigen with high affinity (Pons et al., 2002; Davila et al., 2022). Antibodies are widely used for both disease diagnosis and treatment.

Traditional therapeutic antibody discovery approaches utilized animals, usually mice, to generate polyclonal antibodies against a target antigen. In this approach, candidate monoclonal antibodies (mAbs) are selected and engineered to minimize immunogenicity in humans, while maintaining target specificity and desired pharmacokinetics. The first blockbuster therapeutic antibody (anti-CD3 OKT3), which was engineered in this manner, was approved by the FDA in 1986. Animal-based antibody discovery had a huge impact on the pharmaceutical industry through the 1990’s and motivated the development of new antibody discovery platforms. By the mid-2000’s, approximately one-half of therapeutic antibodies were fully human through the use of transgenic mice or phage display platforms utilizing human BCR genes (Nelson et al., 2010; Ju et al., 2020).

In the past decade, a number of technological breakthroughs have enabled the discovery of antigen-specific mAbs directly from human donors (Pedrioli and Oxenius, 2021). Up to the mid-2000s, mining human B cell receptor (BCR) repertoires for mAbs specific to an antigen of interest was primarily done in academic research labs (Truck et al., 2015; Wang et al., 2015; Goldstein et al., 2019). However, the COVID-19 pandemic brought with it an urgent need for creative ways of targeting the SARS-CoV-2 virus quickly. Remarkably, within months of the pandemic, multiple research groups reported the discovery of neutralizing antibodies from the BCR repertoires of COVID-19 patients (Cao et al., 2020; Hansen et al., 2020; Ju et al., 2020; Pinto et al., 2020; Robbiani et al., 2020; Seydoux et al., 2020; Wang et al., 2020; Zost et al., 2020; Baum et al., 2021). Due to the overwhelming need for a response to the pandemic, along with the rapid availability of resources for COVID-19 related research, many of the mAbs were quickly tested for safety and efficacy in the clinic. The Antibody Society currently lists 35 anti-SARS-CoV-2 mAbs or mAb cocktails undergoing clinical trials (https://www.antibodysociety.org/covid-19-biologics-tracker).

Although it is important not to over-generalize the development of anti-SARS-CoV-2 antibodies to other disease areas, the intensity of research on COVID-19 has refocused attention on the technological innovations that enabled the discovery of antigen-specific antibodies from human BCR repertoires so quickly. Here we review four main areas of innovation: B Cell sorting, BCR sequencing, BCR repertoire analysis, and experimental validation of antigen binding. Although each of these areas are active research topics on their own, the greatest impact on the pharmaceutical industry will come through synthesis into integrated experimental and computational pipelines. Given the recent breakthroughs in computational biology, including antibody-specific machine-learning methods (Akbar et al., 2022), we can expect rapid growth in this area as data generation merges with data analysis in the context of antibody discovery.

## 2 B cell sorting

A repertoire of BCRs refers to a snapshot of all the B cells produced in a given donor at a given time. When studying repertoires, separating cells of interest by cell sorting is commonly used for isolating natural B cells with a specific phenotype or antigen specificity. This is one of the first steps in discovering antibodies from human donors. Common methods used for cell sorting include FACS (Fluorescence-activated cell sorting), MACS (Magnetic-activated cell sorting), or combinations of both. In FACS, fluorescently-labeled antigens are used as probes to isolate antigen-binding B cells, collect them into tubes or plates, and continue further processes such as bulk or single cell BCR gene amplification and sequencing (Gieselmann et al., 2021). Fluorescent-labeling of an antigen is a critical step and can be done *via* covalent chemical conjugation, expression of a recombinant antigen-fluorescent fusion protein, or by biotinylating the antigen and adding fluorochrome-conjugated streptavidin to make an antigen tetramer, which increases avidity to the antibody. It must be kept in mind that such labeling may occlude some part of the epitope and potentially disturb the B cell antigen recognition process (Boonyaratanakornkit and Taylor, 2019). Despite this potential complication, utilization of fluorescent-labeled antigens is a promising approach to the collection of antigen-specific B cells.

A relatively new technology, MACS, utilizes direct (primary antibody-conjugated microbeads) or indirect magnetic labeling (primary antibody plus a secondary antibody-conjugated microbead) of cells prior to separation through a magnetic field. Although it lacks sensitivity and is not compatible with multiple-marker profiles, the cell throughput, viability, and time requirements for MACS are comparable to FACS (Sutermaster and Darling, 2019). Some researchers combine these two methods to do enrichment of antigen-specific B cells (Galson et al., 2015a; Banach et al., 2022).

Isolating antigen-specific human B cells is nevertheless quite challenging. Memory B cells express large amounts of antigen receptors on their surfaces but are present in very low numbers in peripheral blood, the most accessible repertoire compartment of the human body (Waltari et al., 2019). The other most commonly-studied subset of antigen-specific B cells consists of antibody-secreting cells (ASCs). ASCs can be found in higher numbers in peripheral blood, especially after vaccination or infection; however, ASCs, especially those of the IgG isotype, are thought to have limited immunoglobulin surface expression. This might be a reason why many previous studies of ASCs did not utilize antigen-based sorting, but rather collected all ASCs and screened for antigen-specificity downstream after culturing the cells *in vitro* and stimulating antibody secretion before sequencing (Lavinder et al., 2014; Galson et al., 2015a; Acquaye-Seedah et al., 2018; Pedrioli and Oxenius, 2021). However, IgA and IgM isotype ASCs retain expression of surface immunoglobulin (Pinto et al., 2013; Blanc et al., 2016), making it relatively straightforward to sort these subsets in an antigen-specific manner.

**FIGURE 2:**
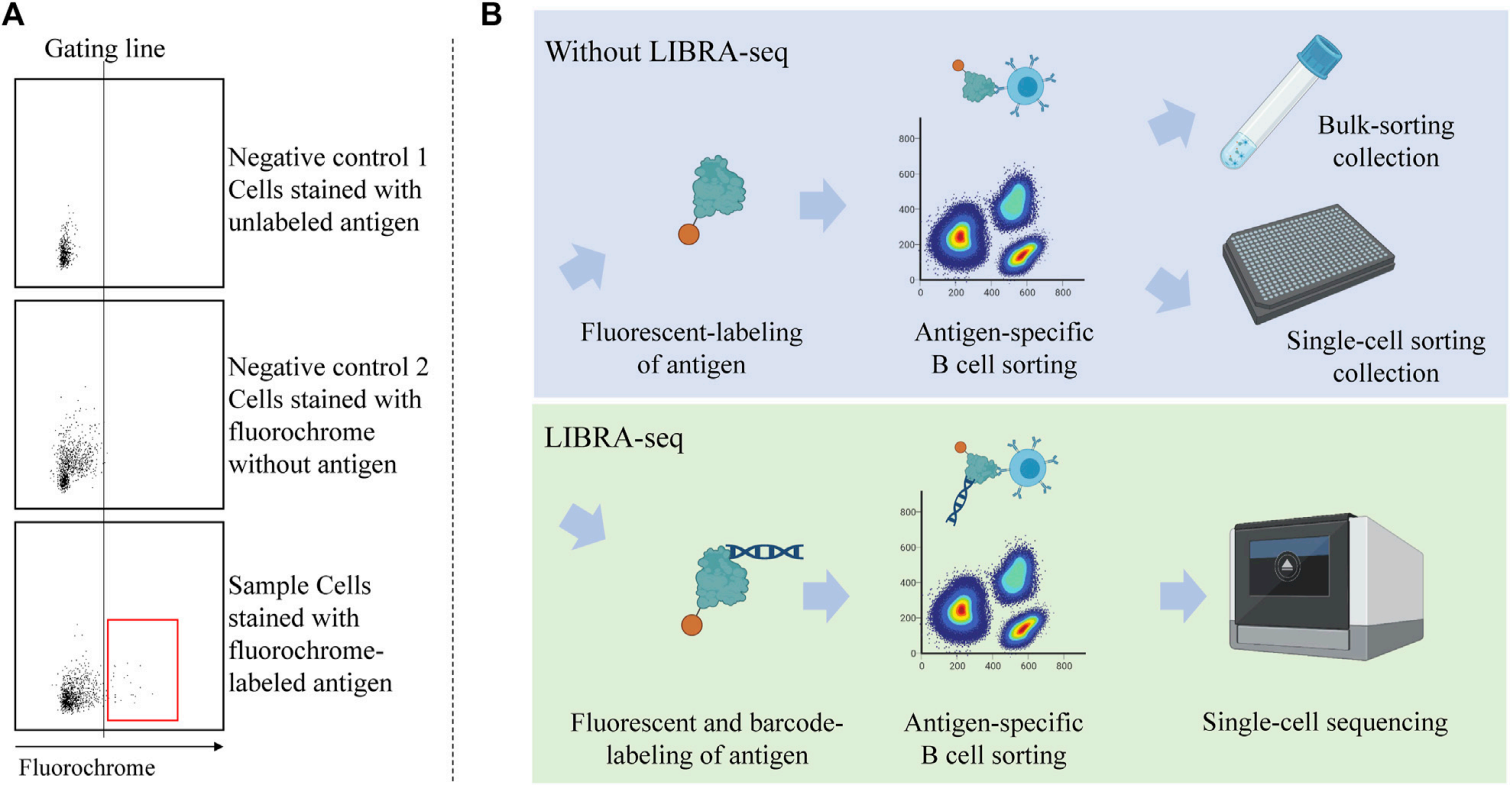
Antigen-specific B cell sorting with or without LIBRA-seq. **(A)** Utilization of double negative control population to determine gating line for selecting antigen-binding cells. The population inside the red box is considered “antigen-binding.” **(B)** Workflow of antigen-specific cell sorting with and without the utilization of LIBRA-seq. Samples can be obtained from vaccinated donors or patients with a certain disease. LIBRA-seq uses barcoded antigen along with the fluorescent label, that can be read by the NGS machine.

Another challenge in antigen-based cell sorting is related to the specificity that is, whether or not the selected cells are truly positive binders or just appear through nonspecific binding to the fluorochrome, streptavidin, or any added linkers (Doucett et al., 2005; Boonyaratanakornkit and Taylor, 2019). During the sorting process, it is necessary to reduce these background signals as much as possible. Due to the limitation of sample quantity and a low number of antigen-specific cells in the sample, we often lack ideal positive control cells from which a positive threshold for fluorescence (gate) can be used to define antigen-binding cells. Thus, in general, one must rely on a negative control population (which can be cells or decoys that are stained by unlabeled antigen or fluorochrome without the antigen) to set the gate for antigen binders (Figure 2A). Additionally, to increase specificity, a double fluorescent staining strategy can be used to label the antigen probe. Here, an antigen is labeled with two different fluorescent labels and the double positive cells are deemed positive (Amanna and Slifka, 2006). However, this approach still leaves some opportunity for non-binders to be recruited, as seen in a previous report that only 80% of sorted cells were positively bound to the antigen after being recombinantly produced and tested by ELISA (Attaf et al., 2020). This observation suggests that experimental validation (discussed in Section 5) is a requirement for any antibody discovery workflow based on antigen-based B cell sorting. This can be a potential bottleneck, since the production of recombinant antibodies following the acquisition of antibody sequences by conventional cloning and expression in mammalian cells can be labor intensive (Pedrioli and Oxenius, 2021).

To overcome these challenges, and to increase the throughput of antigen-based sorting, the Linking B-cell Receptor to Antigen Specificity through Sequencing (LIBRA-seq) method was introduced in recent years (Setliff et al., 2019). LIBRA-seq is a modification of antigen-based cell sorting that makes use of next-generation sequencing (NGS) technology. In LIBRA-seq, in addition to the fluorescent label, the antigen probe is coupled to a unique DNA barcode that is readable in the sequencing stage. The B cells are thus enriched for antigen-binding cells by FACS; then, the specific antigen is mapped to the B cell by the expression level of the barcode (Figure 2B). This allows simultaneous capture of several antigen probes, tagged by the same fluorescent color but different barcodes. Each cell will have scores for each antigen in the screening library. These scores are a function of the unique molecular identifiers (UMIs) for the respective antigen barcodes (Setliff et al., 2019). Several studies utilized this method to efficiently discover SARS-CoV-2 specific antibodies (He et al., 2021; Kramer et al., 2021; Shiakolas et al., 2021; Kramer et al., 2022; Shiakolas et al., 2022; Suryadevara et al., 2022). The latest version of LIBRA-seq allows epitope mapping by barcoding several variants of the antigen, each with known epitopes mutated (Walker et al., 2022).

## 3 B cell receptor sequencing

Due to the unique phenomenon of gene rearrangement in the generation of BCR coding sequences, BCR diversity at the amino acid sequence level is believed to be in the range of 10^16^–10^18^ (Briney et al., 2019). With the development of NGS, High-throughput sequencing-based (HTS) sequencing has been used to analyze both T cell receptor (TCR) and BCR repertoires (Yaari and Kleinstein, 2015). The first use of HTS technology for immune repertoire analysis was made by Campbell (Campbell et al., 2008) in 2008 using the Roche454 platform to explore IGH hypermutation variants carried in patients with chronic B-lymphocytic leukemia at the DNA level. Since this time, a number of new technologies have emerged. These can be divided roughly into two groups: bulk and single-cell sequencing. In bulk sequencing the pairing between heavy and light chains is lost; in single-cell sequencing, this pairing is maintained.

### 3.1 Bulk B cell receptor sequencing

Bulk sequencing provides in-depth information on the frequency of single chains, which gives a high-resolution view of diversity (a measure of the range and distribution of certain features within a given population (Xu et al., 2020)) and clonal expansion (the proliferation of lymphocytes activated by clonal selection in order to produce a clone of identical cells (Polonsky et al., 2016)), as entire cell populations can be sequenced in a single pipeline (Kovaltsuk et al., 2017). Two starting materials can be used as initial templates for repertoire sequence: genomic DNA (gDNA) and messenger RNA (mRNA). gDNA has the advantage of stability and a constant initial gene copy number between cells (Chaudhary and Wesemann, 2018). mRNA as a template requires reverse transcription, during which UMIs can be added, a step that helps in identifying duplicate or/and cloned sequences generated by PCR, which circumvents PCR bias or sequencing errors (Turchaninova et al., 2016; Rosati et al., 2017). In addition, synthetic repertoires utilize long (∼500 bp) oligonucleotide synthesis and high-throughput sequencing to generate a template for every possible V/J combination for minimization of PCR amplification bias, and additional computational normalization to remove residual bias. (Carlson et al., 2013). Multiplex-PCR (m-PCR) and 5′RACE approaches are the two main methods used for amplification. m-PCR has the advantage that only one-step PCR is required, regardless of whether adaptors are included in the primers or not; when the material selected is gDNA, the downstream primers are restricted to several J gene segments, due to the existence of introns (Bashford-Rogers et al., 2014). 5′RACE requires only one set of oligonucleotides, and designing primers from the C gene increases specificity and greatly reduces PCR bias (Yeku and Frohman, 2011), but has a relatively complex workflow for the library building. In addition to BCR information, it is often desirable to obtain the phenotypes or specific subsets of the B cells. Information on immune receptor libraries can be extracted from RNA-seq data, as BCRs are part of bulk RNA-seq data. However, the sensitivity of such an approach is low because of the under-expression of genes at the transcriptional level and also because large-scale RNA-seq usually results in a mixture of cellular gene expression profiles in the sample. Therefore, RNA-seq usually requires pre-targeted protein labeling of cells with fluorescently labeled antibodies to purify the cell types in the sample (Picot et al., 2012).

### 3.2 Single-cell B cell receptor sequencing

To obtain paired heavy-light chain sequences, single B cell resolution is required, since the mRNAs of each chain are physically separate. When coupled with single-cell RNA-seq, BCR sequencing can also provide important phenotype information on the cells (Haque et al., 2017). Goldstein and co-workers showed that single B cell sequencing can recover a higher number of antibody lineages compared to hybridoma technology (Goldstein et al., 2019). Single-cell sequencing has also been used to identify SARS-CoV-2 specific Abs (Woodruff et al., 2020; He et al., 2021), tumor-specific Abs (Buus et al., 2021), and autoimmune disease-specific Abs (Sulen et al., 2020; Jin et al., 2021). Single-cell sequencing is now readily available from several companies, including 10x Genomics and Takara Bio.

Single-cell sequencing combines multiple levels of information, not limited to intracellular gene expression and BCR pairing information; by adding specific oligonucleotide barcode-associated antibodies, thereby allowing surface proteins to be characterized, similar to flow cytometry. Single cells can be isolated in microtiter plates or droplets and then physically linked by overlapping extension RT-PCR in the variable regions of heavy and light chains. Although the potential to obtain BCR pairing information at high throughput has been demonstrated, this technique requires custom equipment and does not yield full-length variable region sequence information (Goldstein et al., 2019). Although full-length sequences can be inferred by the assembly, there is uncertainty in this process (DeKosky et al., 2013; DeKosky et al., 2015; McDaniel et al., 2016). RAGE-seq (repertoire and gene expression by sequencing) combines the genomic technologies of Oxford Nanopore Technologies’ long reads, 10x Genomics, Illumina’s short reads, and CaptureSeq4 major platforms to enrich RNA from single B cell, and then assembles full-length sequences computationally (Singh et al., 2019).

The main limitation to current single-cell sequencing is the tradeoff between sequencing depth and cost. Sequencing depth is the number of transcripts detected from each cell which should be controlled together with the number of cells to get enough coverage (average number of reads that align to specific locus in a reference genome to “cover” reference bases) for confident sequence assignment. The minimum sequencing depth for single cell VDJ analysis is around 5,000 paired reads per cell, while gene expression analysis requires a minimum 20,000 reads per cell, which can be increased depending on needs to analyze a greater number of genes. In comparison with bulk sequencing, which usually obtains 1 million reads per sample, singe-cell sequencing depth depends on the desired number of cells in one sample. For example, we recently obtained approximately 20 million reads from one thousand cells with 20,000 reads per cell (unpublished results). The cost of single-cell sequencing mainly comes from the library preparation step which can be 10–20 times higher than for traditional bulk sequencing. Current developments are focused on how to reduce the cost and increase the sequencing depth of single-cell sequencing (Haque et al., 2017; Upadhyay et al., 2018; Wu et al., 2018).

### 3.3 Annotation of raw sequence data

Annotation includes defining the V, D, and J genes for a given BCR, inferring the accurate amino acid sequence, and assigning the CDR boundaries. These are nontrivial tasks. Several numbering schemes to define CDRs have been proposed including Kabat, Chothia, Martin, Gelfand, IMGT, and AHo (Dondelinger et al., 2018). Meanwhile, several tools have been developed to streamline the process of annotation to use these numbering schemes. For the assignment of CDRs ANARCI is a reliable and user-friendly tool (Dunbar and Deane, 2016). For gene and amino acid assignment, the strengths of the various tools have been systematically discussed in several previous publications (Heather et al., 2018; Lopez-Santibanez-Jacome et al., 2019; Smakaj et al., 2020). Here, we will describe several of tools for the analysis of bulk- and single-cell sequence data. IMGT is one of the most widely used annotation platforms today. High-quality germline sequence information for most species is assembled in IMGT, and therefore reference libraries for the vast majority of sequence annotation tools are derived from IMGT (Lefranc et al., 2005; Manso et al., 2022). In 2011, IMGT developed a platform for HTS T/B repertoire data, supporting raw sequence uploads in FASTA and FASTQ formats (Alamyar et al., 2012; Li et al., 2013). IgBLAST was originally developed as a tool for analyzing immunoglobulin sequences using BLAST, a local alignment method (Ye et al., 2013). Although data can be uploaded directly through the webpage, it does not show many advantages in the analysis of HTS data or presentation of results. MiXCR is another widely used stand-alone package for BCR repertoire annotation, as there is no restriction on the number of sequences. It uses an improved k-mer chaining algorithm for sequence alignment, and an error correction procedure can be performed based on the quality of the sequences (Bolotin et al., 2015).

For scRNA-seq data generated through the 10x Genomics platform, pre-processing similar to bulk sequencing is required before downstream analysis. The company offers Cell Ranger (Zheng et al., 2017), which is recommended for its ability to process both gene expression and paired TCR/BCR data. The development of tools to reconstruct immune repertoire information from single-cell or bulk RNA-seq is an active area, including tools such as BASIC, BRACER, and BALDR (Haque et al., 2017; Upadhyay et al., 2018; Wu et al., 2018).

## 4 B cell receptor repertoire analysis

BCR repertoire sequence data is growing rapidly. In this section, we will first describe methods to analyze diversity, clonal composition, or the specificity of BCRs from different cohorts. Next, we introduce databases and platforms to store repertoire data. Lastly, we will briefly discuss antibody structural modeling and epitope/paratope prediction.

### 4.1 B cell receptor repertoire sequence analysis

**FIGURE 3:**
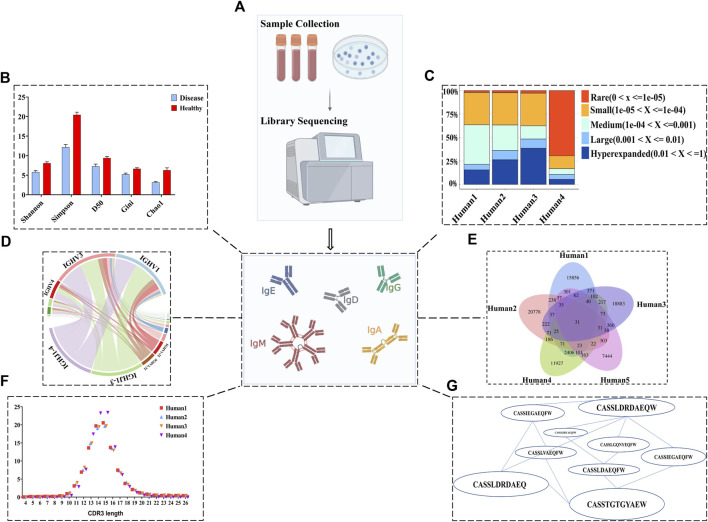
BCR repertoire sequence analysis. **(A)** Sample collection using PBMCs from blood, followed by NGS. **(B)** The Shannon, Simpson, D50, Gini, and chao1 indices are designed to assess the overall diversity of each cohort. **(C)** Clone proportion can show the change of high-frequency (clone expansion) as well as low-frequency sequences in each sample. **(D)** The bias of V and J gene usage and their combination can reflect immune responses of different repertoires. **(E)** The length distribution of the CDR3 region can be used to characterize repertoire. **(F)** Venn plot can be utilized for visualizing the degree of convergence among samples and exploring the potentially disease-specific public clone. **(G)** Networks of BCRs.

Sequence analysis of BCR repertoire data is a rapidly evolving field that can be roughly divided into three main components: diversity, clonal composition (the relative abundance of specific clones (ImmunoMind, 2019)), and antigen/disease binding specificity. There are a large number of tools and packages that can be used to analyze BCR diversity, clonal frequency, and networks of BCRs. Some well-used tools like Immcantation (Vander Heiden et al., 2014; Gupta et al., 2015), and Immunarch (ImmunoMind, 2019) allows visualization of results after direct import of data from Cell Ranger or MiXCR. Some representative visualizations of results are shown in Figure 3. Many metrics in repertoire analysis are general methods used beyond BCRs or TCRs, such as Shannon and Simpson diversity (Leinster and Cobbold, 2012; Greiff et al., 2015a). Shannon diversity correlates with increasing sequence uniformity, whereas Simpson diversity assigns greater weight to dominant sequences. Clonal abundance is another measurement to quantify sequence distribution that can be used to determine the difference in the ratio of high-frequency to low-frequency sequences between healthy and diseased patients (Yaari and Kleinstein, 2015). High expression or low expression of V, D, and J BCR genes can indicate immune responses (Lee et al., 2021; Kotagiri et al., 2022). Meanwhile, the length and amino acid usage of CDR3 regions is often used to characterize repertoires in terms of a few dependent parameters. For example, one study reported that the average CDRH3 lengths of IGHG1, IGHA1, and IGHA2 were significantly greater in COVID-19 patients than healthy cohorts (Galson et al., 2020). Moreover, the same authors identified CDRH3 amino acid sequence signatures within COVID-19 patients with different symptoms (Galson et al., 2020). Also, diversity caused by high-frequency mutations in somatic cells is another important feature of BCR sequences. In general, mutation analysis shows the extent of differentiation compared to germline sequences and indicates antigen-driven affinity maturation. One group utilized the frequency of somatic hypermutation (SHM) of the heavy chain as a feature to identify HIV patients possessing broadly neutralizing antibodies (Roskin et al., 2020).

Identification patterns relating to specific antigens or disease cohorts is a major challenge in BCR repertoire analysis. Previous studies have observed BCR sharing among HIV patients, HBV vaccination donors, Influenza vaccination donors, and COVID-19 patients (Jackson et al., 2014; Galson et al., 2015a; Setliff et al., 2018; Kim et al., 2021; Voss et al., 2021). In order to quantify the convergence (the existence of similar or identical BCR sequences among donors in a common cohort) of BCRs, clonotypes (same V, J gene and identical amino acids in CDR3 region) analysis is widely used in both bulk and single-cell analyses (Soto et al., 2019; Raybould et al., 2021b). Furthermore, clustering such clonotypes (e.g. with 80% or greater sequence identity in their CDR3) is often used (Galson et al., 2020; Nielsen et al., 2020). Similar CDR3 sequences that dominate the immune response in different individuals following antigen stimulation are often referred to as a “convergent” or “public” (Truck et al., 2015). Experimental validation of clustering will be described in detail in section 5.2.

### 4.2 Repertoire databases and data mining

Due to continuous advances in sequencing technology, BCR repertoire sequence data, especially bulk data, has grown rapidly in recent years. Most data associated with published repertoire research is stored in public databases such as the Sequence Read Archive (SRA) or European Nucleotide Archive (ENA), in the form of raw NGS reads. Since SRA and ENA do not allow sequence-level searches, such analysis must be performed on specialized repertoire web servers or by using command-line tools. In this section, we describe a number of databases for antibody sequences, structures or both, that can help in the mining of antigen-specific BCR sequences.

**FIGURE 4:**
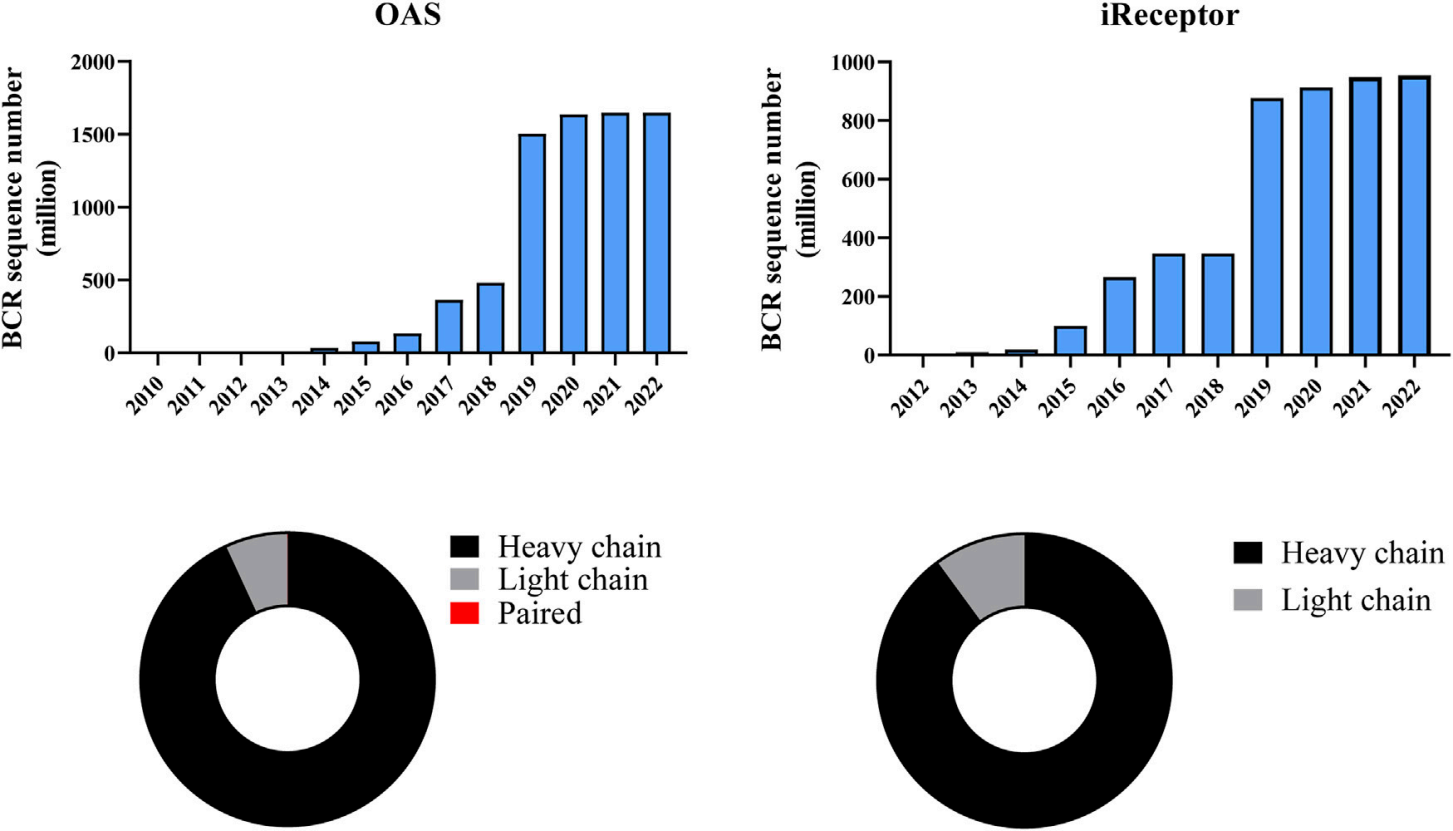
Metadata available in OAS and iReceptor. The bar chart shows the cumulative growth of BCR sequence data. OAS provides about 1,650 million and iReceptor provides about 955 million BCR sequences (data updated on 1 September 2022). The donut graph shows the ratio of chain type in each database. Most BCR sequences are heavy chains in both OAS (93%) and iReceptor (90%). Only 0.00011% sequences in OAS are paired BCRs, which are not visible in the donut graph.

• Observed Antibody Space (OAS) is a comprehensive and frequently updated website and database (Kovaltsuk et al., 2018; Olsen et al., 2022) Although OAS also contains paired data, to our knowledge, it is the first organized collection of bulk BCR sequences. Metadata such as study, species, disease, vaccine, B cell source, and subset can be searched (Figure 4).
• The iReceptor (Corrie et al., 2018) platform allows sharing and comparing adaptive immune receptor repertoire (AIRR)-seq data. It has two key components: a data repository that focuses on AIRR data, and a web-based Scientific Gateway that allows researchers to discover, federate, explore, and analyze AIRR-seq data (Figure 4).
• SAbDab (Dunbar et al., 2014) is a frequently updated resource containing all publicly available antibody structures and, similar to OAS, is convenient to search using metadata, including species, experimental method, resolution, or amino acid at a given position using canonical numbering.
• IMGT/3Dstructure-DB (Ehrenmann et al., 2010) is a three-dimensional structure database of IMGT entries that stores the structures of immunoglobulins, TCRs, and major histocompatibility complex proteins of humans and other vertebrate species. A related database, IMGT/2Dstructure-DB, stores the amino acid sequences from INN/WHO and Kabat databases (Ehrenmann and Lefranc, 2011). IMGT/3Dstructure-DB contains 8,437 entries as of 3 June 2022.
• huARdb (Wu et al., 2022) is a versatile and user-friendly web interface consisting of data from 444,794 high confidence T or B cells with full-length TCR/BCR sequences and transcriptomes from 215 datasets, which have been subjected to a uniform workflow.
• PIRD (Zhang et al., 2020) is a multi-species BCR dataset that contains 5 main information modules, including project information, sample information, raw sequencing data, annotated TCR or BCR repertoires, and a database of TCRs and BCRs targeting known antigens (TBAbd). PIRD can also carry out analyses, including biased gene usage, the length distribution of CDR3, and the diversity index for each dataset directly.
• ImmPort (Bhattacharya et al., 2018) is one of the largest repositories of open immunology data. It hosts data from more than 300 clinical and mechanistic studies in humans and immunological studies on model organisms, categorized as Private Data, Shared Data, Data Analysis, and Resources, with a focus on allergy, autoimmune disease, infection response, transplantation, and vaccine response.
• cAb-Rep (Guo et al., 2019) contains 306 immunoglobulin repertoires from a database consisting of 121 healthy, vaccinated, or autoimmune disease donors. The database contains 267.9 million IGH and 72.9 IGL full- or nearly full-length transcripts that have been annotated according to isotype, somatic hypermutation (SHM), and other biological characteristics.
• ImmuneDB (Rosenfeld et al., 2017; Monasterio et al., 2018) is a high-throughput immune receptor sequencing data system that integrates data storage and analysis. The developers demonstrated that ImmuneDB and MiXCR have comparable performance in annotating raw data. Output includes selection pressure, lineage mapping, novel allele detection, etc. ImmuneDB states that their method can quickly identify more potential sequences compared with IMGT/High-Vquest and that IT performs similar to MiXCR on the same input data.
• VDJServer (Christley et al., 2018) integrates large data storage and analysis. The advantage of VDJServer is that sequence annotations can be performed after quality control screening of raw data.
• Our lab has recently launched InterClone (Wilamowski et al., 2022), a resource that contains both BCR and TCR repertoire data, along with tools to store, search or cluster the data. Distinguishing features of InterClone include: the ability of users to control the visibility of their data; efficient encoding of CDR regions to allow flexible searches or clustering using user-specified similarity thresholds for CDRs; a large amount of BCR data, particularly for COVID-19, influenza, HIV, and healthy donors.
• There are also databases created for specific diseases. CoV-AbDab (Raybould et al., 2021a) currently contains 10,005 antibodies and nanobodies from published papers/patents that bind to at least one betacoronaviruses (last updated: 26th July 2022). This database is the first known integration of antibodies that bind SARS-Cov2 and other betacoronaviruses, including SARS-CoV1 and MERS-CoV. It contains evidence of cross-neutralization, the origin of antibody nanobodies, full-length variable structural domain sequences, germline assignments, epitope regions, PDB codes (if relevant), homology models, and literature references.
• CATNAP (Yoon et al., 2015) is a web server for HIV data, including antibody sequences from the authors’ own and published studies. As input, users can select specific antibodies or viruses, a panel from published studies, or search using local data. The output overlays neutralization panel data, viral epidemiology data, and viral protein sequence comparison on a single page with further information and analysis. Users can highlight alignment positions, or select antibody contact residues and view position-specific information from the HIV database.

### 4.3 Antibody structure prediction

Protein structure prediction is one of the areas of computational biology that has progressed most rapidly in recent years, owing to breakthroughs in Deep learning (Baek et al., 2021; Jumper et al., 2021). Before these breakthroughs, a plethora of antibody modeling tools existed that performed similarly well for all regions except CDRH3 loops (Almagro et al., 2014). However, it is likely that going forward, all state-of-the-art methods for antibody modeling will utilize some aspects of current Deep learning-based protein structural modeling methods. An important first step in this direction is DeepAb, which convincingly out-performed traditional template-based methods, including our own Repertoire Builder, in terms of antibody structural accuracy (Ruffolo et al., 2022). In a recent assessment, we found that the average CDRH3 root-mean-square deviation (RMSD) dropped from 4.38 to 3.44 Å for AlphaFold compared with our own, previously state-of-the-art, Repertoire Builder in a large and diverse set of 620 antibodies (Xu et al., 2022). Therefore, for antibodies without bound antigens, the current Deep learning approach appears to be a significant improvement.

Unfortunately, it is becoming accepted that the multi-chain extension of AlphaFold, AlphaFold multimer, does not work well for antibody-antigen complexes (Evans et al., 2022). This problem probably arises in part from the fact that AlphaFold uses overall sequence similarity to construct multiple sequence alignments, which are, in turn, used as feature vectors. Many antibodies that target different antigens will be aligned in this process, resulting in a noisy signal. Indeed, when we assessed the complex modeling performance of AlphaFold multimer using a small benchmark of 25 antibody-antigen complexes, we found that the vast majority were docked to the wrong epitope (Standley et al., 2022). Therefore, it will be interesting to see if a more careful selection of sequences and structural templates within the Deep learning workflow will lead to more coherent antibody-antigen complex modeling. CDR-based clustering is one of the functions repertoire databases (see Section 4.2) can provide. Another interesting direction is to couple antibody-antigen complex modeling with epitope prediction.

### 4.4 Epitope prediction

As of 19 July 2022, there were only 9,811 recorded antibody-antigen structures available in the Protein Data Bank (Berman et al., 2008; Raybould et al., 2020). Due to the time-consuming and labor-intensive process of experimental methods to investigate antibody-antigen interactions experimentally (see Section 5), there is a need for computational approaches that can quickly predict the epitope and paratope from sequence or structure information. Compared to the difficulty of epitope prediction (where almost any surface patch of antigen could be an epitope for some antibody), the paratope prediction problem is relatively easy. Most paratopes are located within the six CDRs in the variable fragment of heavy and light chains. Many published tools like Parapred (Liberis et al., 2018) and proABC-2 (Ambrosetti et al., 2020) can achieve satisfactory performance in paratope prediction. Thus, in this section, we will focus on epitope prediction.

In recent decades, many tools have been developed in order to predict continuous/linear B-cell epitopes using antigen sequence information or discontinuous/conformational B-cell epitope using antigen structure information. These methods generally adopt machine learning approaches (support vector machines, random forests, linear regression, and neural networks) to learn epitope features from known complex structures (Table 1). One problem with many epitope prediction tools is that they only use features of the antigen, whereas we are generally interested in antibody-specific epitopes (Sela-Culang et al., 2015). The direction to solve this problem is to introduce antibody features into the process of epitope prediction. Some tools, including PECAN (Pittala and Bailey-Kellogg, 2020) and Pinet (Dai and Bailey-Kellogg, 2021) used Deep learning to extract antibody and antigen features for use in epitope prediction. Other tools, like EpiPred (Krawczyk et al., 2014), MAbTope (Bourquard et al., 2018), and AbAdapt (Davila et al., 2022), incorporate antibody-antigen docking-based features; these studies have demonstrated that the inclusion of the antibody features improves epitope prediction. As expected, antibody-antigen docking is sensitive to antibody model quality (Davila et al., 2022). We recently incorporated the more accurate antibody models produced by AlphaFold (Jumper et al., 2021) into the AbAdapt pipeline (Xu et al., 2022). We observed significant improvement in docking, paratope prediction, and antibody-specific epitope prediction compared with the default AbAdapt pipeline. In a realistic case, using an anti-SARS-CoV-2 RBD antibody complex benchmark, the use of AlphaFold resulted in higher epitope prediction accuracy than all other tested tools.

**TABLE 1:** Summary of the epitope prediction tool.

Catalog	Names	Availability	Method	References
linear B-cell epitope	ABCPred	https://webs.iiitd.edu.in/raghava/abcpred/index.html	Recurrent neural network	Saha and Raghava (2006)
linear B-cell epitope	AAPred	https://www.bioinf.ru/aappred/	Support vector machine	Davydov and Tonevitsky (2009)
linear B-cell epitope	FBCPred/BCPREDS	https://ailab.cs.iastate.edu/bcpreds/	Two machine learning approaches	El-Manzalawy et al. (2008)
linear B-cell epitope	COBEpro	https://scratch.proteomics.ics.uci.edu	Support vector machine	Sweredoski and Baldi (2009)
linear B-cell epitope	BepiPred-2.0	https://services.healthtech.dtu.dk/service.php?BepiPred-2.0	Random forest	Jespersen et al. (2017)
linear B-cell epitope	Lbtope	http://crdd.osdd.net/raghava/lbtope/	Support vector machine and k-nearest neighbor	Singh et al. (2013)
linear B-cell epitope	DRREP	https://github.com/CorticalComputer/DRREP	Deep neural network	Sher et al. (2017)
linear B-cell epitope	SVMTriP	http://sysbio.unl.edu/SVMTriP	Support vector machine	Yao et al. (2012)
linear B-cell epitope	LBEEP	https://github.com/brsaran/LBEEP	Support vector machine and AdaBoost-random forest	Saravanan and Gautham (2015)
linear B-cell epitope	EPMLR	http://www.bioinfo.tsinghua.edu.cn/epitope/EPMLR/	Multiple linear regression	Lian et al. (2014)
linear B-cell epitope	iBCE-EL	http://thegleelab.org/iBCE-EL	Randomized tree and gradient boosting classifiers	Manavalan et al. (2018)
linear B-cell epitope	iLBE	http://kurata14.bio.kyutech.ac.jp/iLBE/	Random forest	Hasan et al. (2020)
linear B-cell epitope	EpiDope	http://github.com/mcollatz/EpiDope	Deep neural network	Collatz et al. (2021)
Conformational B-Cell epitope	EliPro	http://tools.iedb.org/ellipro/	Clustering of neighboring residues based on protrusion index	Ponomarenko et al. (2008)
Conformational B-Cell epitope	PEPITO	http://pepito.proteomics.ics.uci.edu/	Linear combination	Sweredoski and Baldi (2008)
Conformational B-Cell epitope	CBTOPE	http://www.imtech.res.in/raghava/cbtope/	Support vector machine	Ansari and Raghava (2010)
Conformational B-Cell epitope	DiscoTope 2.0	https://services.healthtech.dtu.dk/service.php?DiscoTope-2.0	Epitope propensity scores	Kringelum et al. (2012)
Conformational B-Cell epitope	SEPPA 3.0	http://www.badd-cao.net/seppa3/index.html	Logistic regression and clustering coefficient	Zhou et al. (2019)
Conformational B-Cell epitope	CluSMOTE	https://github.com/BSolihah/conformational-epitope-predictor	Support vector machine and decision tree	Solihah et al. (2020)
Combining antibody feature	EpiPred	http://opig.stats.ox.ac.uk/webapps/newsabdab/sabpred/epipred/	Combing the conformational matching of structures and a specific score	Krawczyk et al. (2014)
Combining antibody feature	MAbTope	Lead corresponding contact	Integration of docking-based prediction method and experimental steps	Bourquard et al. (2018)
Combining antibody feature	PECAN	https://github.com/vamships/PECAN	Paratope and epitope prediction with graph convolution attention network	Pittala and Bailey-Kellogg (2020)
Combining antibody feature	Pinet	https://github.com/FTD007/Pinet	Geometric deep neural network	Dai and Bailey-Kellogg (2021)
Combining antibody feature	AbAdapt	https://sysimm.org/abadapt/	Combining docking-based features to predict antibody-specific epitope	Davila et al. (2022)

It is also worth noting that the combination of different deep or machine learning models is becoming a general trend. A Deep learning framework was developed to extract local features around target residues and global features of the full antigen sequence using Graph Convolutional Networks (GCNs) and Attention-Based Bidirectional Long Short-Term Memory (Att-BLSTM) networks separately (Lu et al., 2022). The local and global features from two networks were combined to predict the epitope and demonstrate that global features play a critical role in structure-based epitope prediction (Lu et al., 2022). Moreover, recent work introduced general protein language models that not only focus on the reported antigen-antibody complex to capture binding patterns, but also used the deep transformer based protein language model, ESM-1b (Rives et al., 2021), to achieve more accurate epitope prediction only using the antigen sequence information in BepiPred-3.0 (Clifford et al., 2022). Recently, Robert and co-workers used simulated antibody-antigen data in order to circumvent the lack of experimentally-determined antibody-antigen structure complexes and focused on the challenging problem of learning antigen and epitope specificity features from antibody sequences (Robert et al., 2022). The potential advantage of these later methods is that they circumvent the time-consuming docking step.

## 5 Experimental validation

### 5.1 Epitope discovery

A prerequisite to therapeutic antibody discovery is to identify the epitope for a given antibody-antigen pair. There are several well-established experimental approaches to elucidate the epitope information including X-ray crystallography, nuclear magnetic resonance (NMR), peptide-based microarrays, mutagenesis, and cryo-electron microscopy (Cryo-EM). X-ray crystallography is the gold standard to determine the precise binding between antibody and antigen (Holcomb et al., 2017). However, X-ray crystallography has disadvantages in terms of throughput and cost; moreover, flexible or membrane-bound antigens are notoriously difficult to crystallize (Abbott et al., 2014). Nuclear magnetic resonance can also be utilized to obtain detailed epitope mapping information (Blech et al., 2013). But this method has relatively low sensitivity, requires high purity and solubility, and small size for the proteins (Wüthrich, 1990; Pan et al., 2016). Although peptide-based microarrays are high-throughput, and can sometimes identify epitopes with high sensitivity, peptide-based microarray performance is limited by various factors: affinity of the peptides, immobilization methods, and conformational constraints induced by the immobilization. Furthermore, the limitation of linear epitopes is a major concern (Qi et al., 2019). Mutagenesis allows the investigation of epitopes without the need for structure determination. For example, using the alanine shotgun approach, epitopes for difficult proteins such as membrane proteins can be quickly identified. One disadvantage of this approach is that it is hard to clarify whether the mutation has disrupted the folding (Peng et al., 2011).

Due to the combined requirement to quickly and precisely identify epitopes, many studies combine traditional epitope discovery strategies with cutting-edge technologies. Antibody binding epitope Mapping (AbMap), a combination of phage-displayed peptide libraries with next-generation sequencing, was developed to determine 200 antibody-specific epitopes in a single run (Qi et al., 2021). Additionally, microarrays consisting of 648 overlapping peptides that cover the four major structural proteins of the SARS-CoV-2 virus have been constructed: Spike, Nucleocapsid, Membrane, and Envelope (Hotop et al., 2022). This microarray fingerprint of positive serum samples was learned by a machine learning model and the epitopes were used to diagnose COVID-19 positive and negative donors (Hotop et al., 2022).

Among the wide range of experimental epitope mapping methods, we will focus on two promising approaches: Hydrogen-Deuterium Exchange Mass Spectrometry (HDX-MS) and Deep Mutational Scanning (DMS), which have the potential to perform medium-throughput epitope discovery.

HDX-MS measures changes in the mass of a protein by isotope exchange between amide hydrogens of the protein backbone and its surrounding solvent. The folded state of the protein and its dynamics will affect the rate of this exchange (Masson et al., 2019). In recent years, HDX-MS has been increasingly used for epitope and paratope mapping of antibody-antigen complexes due to its speed and small sample size requirements, and insensitivity to protein size. A semi-automated HDX-MS workflow was used to perform epitope mapping of Fab-CR6261 with diverse influenza Hemagglutinin subtypes (Puchades et al., 2019). Similarly, an in-house HDX-MS system was constructed to explore the binding of birch Bet v1 protein, a native pollen allergen, in the presence of four antibodies that target non-redundant epitopes (Zhang et al., 2018). Two uncontentious epitope loops of TL1A with anti-TL1A monoclonal antibody 1 were identified by HDX-MS (Huang et al., 2018).

**FIGURE 5:**
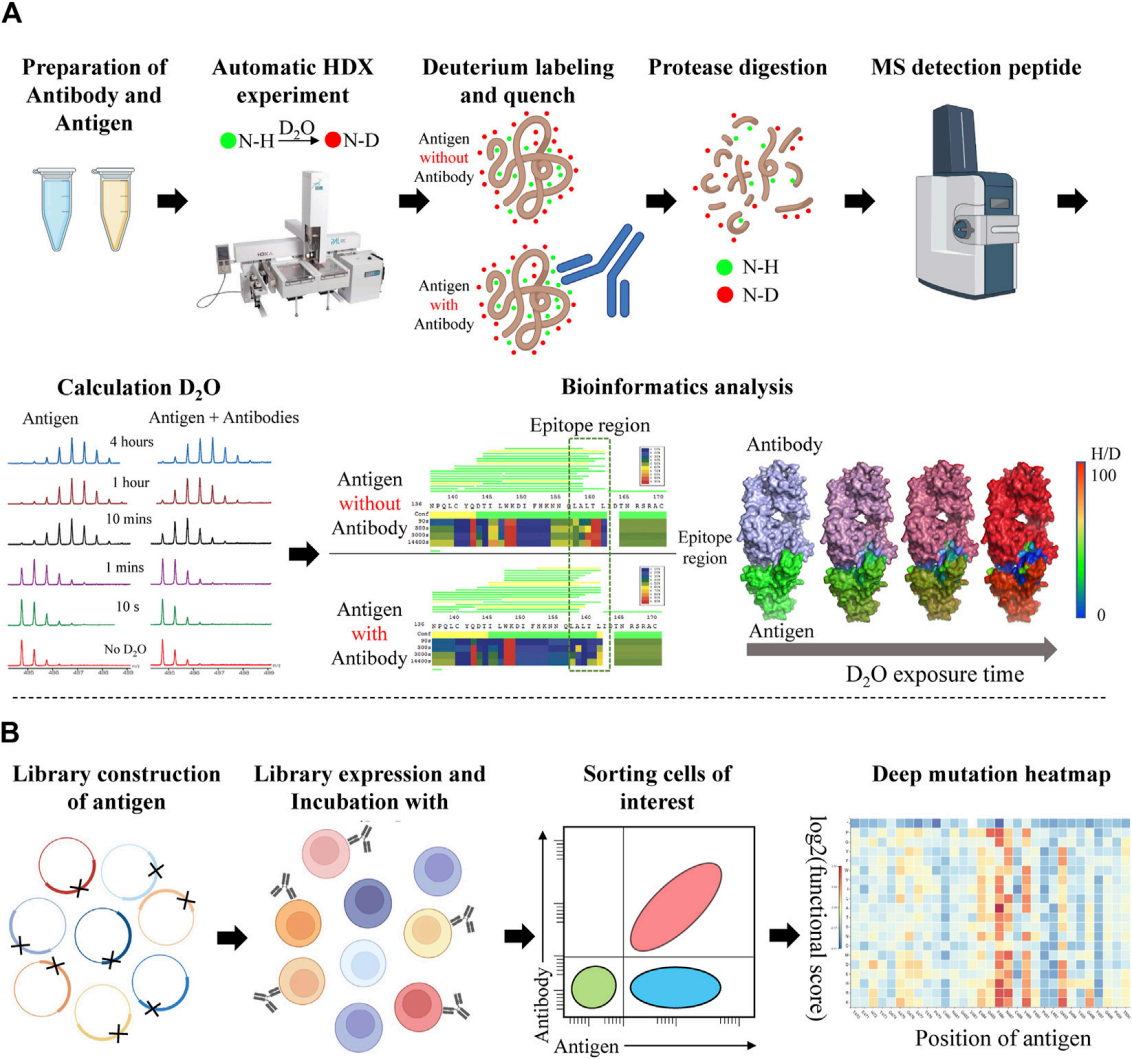
Workflow of Hydrogen-Deuterium Exchange Mass Spectrometry (HDX-MS) and Deep mutational scanning (DMS) for epitope mapping. **(A)** The HDX-MS workflow consists of high-quality protein sample preparation; antigen HDX experiment with or without the presence antibody; antigen peptides are processed by MS; levels of deuteration are quantified by intensity-weighted centroid m/z value; epitope mapping. **(B)** The DMS workflow consists of library construction of antigen mutants; expression; coincubation with antibody; cell sorting; sequencing; visualization of results as a heatmap.

The routine workflow of HDX-MS epitope mapping is performed by using antigen alone as a reference and in the presence of antibodies. The antigen and antibody are labeled in D_2_O buffer under equilibrium conditions at several time points. Compared with antigen alone, the contact of antibody lowers the solvent exposure of antigen residues in the epitope region and leads to the reduction of deuterium incorporation. After protease digestion, proteolytic peptides are desalted and separated on a mass spectrum (MS) system. The fingerprint of antigen alone and antigen-antibody complex will be captured and analyzed by downstream bioinformatics analysis (Figure 5A) (Tran et al., 2022). However, HDX-MS also has some limitations. The major limitation is that HDX-MS can only capture peptide-level information and the individual residue contribution among peptides remains uncertain. Another limitation is the insufficient sequence coverage of peptides spanning the whole protein sequence (Masson et al., 2019). HDX-MS also can’t capture the information of prolines which do not have an amide hydrogen group for deuterium exchange (Huang et al., 2018). Recent studies have incorporated peptide-level information from HDX-MS with antibody-antigen docking to overcome the drawbacks of either method alone (Bennett et al., 2019; Fields et al., 2021).

DMS makes use of massive (typically 1 million) mutant versions of a protein in a single experiment to reveal their intrinsic properties by analyzing large-scale phenotype readouts (Fowler and Fields, 2014). By incorporating NGS, the DMS method can observe the effect of individual mutants in a large population. A typical DMS workflow for epitope mapping includes library construction and mutation design of the antigen; library expression and incubation with antibody; sorting cells of interest by FACS or measuring the binding affinity; sequencing the selected mutations and constructing the data of deep mutation heatmap through bioinformatics analysis. (Figure 5B).

DMS has been used to investigate various disease-related antigens. In one study, DMS was utilized to precisely map the epitopes of a panel of cross-neutralizing nanobodies against H1N1 and H5N1 (Gaiotto and Hufton, 2016). In another study, functional constraints and comprehensive mutations of the Zika virus envelope (E) protein were constructed and the effects of viral growth as well as viral neutralization by two monoclonal antibodies were measured (Sourisseau et al., 2019). Additionally, a platform that combines immunoprecipitation of phage peptide libraries and DMS (Phage-DMS) was constructed. Through Phage-DMS, the authors designed all possible amino acid variants of the HIV Envelope and performed fine mapping of epitopes using four well-characterized HIV antibodies (Garrett et al., 2020). In a recent report, all mutations to the SARS-CoV-2 RBD were first measured by DMS and the effect of expression and affinity for ACE2 were also evaluated (Starr et al., 2020). Meanwhile, DMS was also used to systematically mutate Wuhan-Hu-1, Alpha, Beta, Delta, and Eta variant RBDs and identified some substitutions that cause epistatic shifts during viral evolution (Starr et al., 2022). Also, many studies have reported the utilization of DMS to investigate hotspots of SARS-CoV-2 RBD that enable escape from neutralizing antibodies (Greaney et al., 2021a; Greaney et al., 2021b; Starr et al., 2021; Tsai et al., 2021). These applications convincingly demonstrate that DMS can facilitate the understanding of antigen function and systematically evaluate antibody escape.

In addition to epitope analysis, DMS can be applied to antibodies themselves to identify paratopes or for optimizing other phenotypes. In one case, DMS was used to identify many affinity-enhancing mutations at the variable light-heavy chain interface of an anti-lysozyme antibody; a variant with tenfold higher affinity as well as substantially improved stability were identified (Warszawski et al., 2019). Furthermore, a fully automated design protocol, AbLIFT, was established for improving molecular interactions across the variable light-heavy interface and applied to anti-VEGF/QSOX1 antibodies to improve affinity, stability, and expression (Warszawski et al., 2019). In another application, DMS was combined with Deep learning to optimize the affinity, viscosity, clearance, solubility, and immunogenicity of trastuzumab (Mason et al., 2021). Recently, by leveraging DMS technology, researchers engineered a nanobody initially specific for SARS-CoV-1 RBD in order to bind SARS-CoV-2 RBD (Laroche et al., 2022). Our group contributed to the DMS-based engineering of an ACE2 decoy that could neutralize the SARS-Cov-2 Omicron variant and proved the decoy prevented escape for each single-residue mutation in the RBD of SARS-Cov-2 (Ikemura et al., 2022). We also constructed a database, SpikeDB, that provides changes in infectivity, antigenic escape, ACE2 affinity, and protein expression caused by point mutations in the spike protein of SARS-CoV-2 using DMS (Ikemura et al., 2022).

### 5.2 Validation of repertoire data mining

Repertoire sequencing is generating large amounts of human BCR data. However, most published BCR sequences lack information about targeted antigen or epitope. The development of antigen-specific B cell sorting technologies such as LIBRA-seq could solve the problem of assigning antibodies to their antigens (see section 2) (Setliff et al., 2019). Although antigen-specific B cell sorting is quite a powerful approach, it requires recombinant antigens and specialized cell sorting techniques that are difficult to scale to large numbers of antigens. Computational approaches to identifying antigen-specific antibodies are one way of simplifying very large repertoire sequence data sets.

Various methods are being developed to cluster antibodies that target the same antigen and epitope. Some approaches use information from antibody sequence only, while others use structure information, if available. (Galson et al., 2015b; Xu et al., 2019; Ripoll et al., 2021; Wong et al., 2021). Here, we will focus on sequence-based approaches to search for antibodies with similar target antigens and epitopes.

Clustering of clonotypes was the first method used to group antibodies that possibly target the same antigen (Reddy et al., 2010; Zhu et al., 2013; Galson et al., 2015b; Greiff et al., 2015b). This approach assumes that antibodies with the same V and J genes as well as a given CDR3 amino acid sequence identity (e.g. 80%–100%) in the heavy chain are more likely than other BCRs to target the same antigen and epitope (Galson et al., 2015b; Truck et al., 2015; Soto et al., 2019). Clustering of clonotypes can be applied to single-chain (usually heavy chain) or heavy and light chain paired data (Raybould et al., 2021b). A recent study assembled approximately 8,000 published COVID-19 antibodies from more than 200 donors and demonstrated that antibodies binding to SARS-CoV-2 spike RBD, NTD or S2 possessed distinct convergent clonotype features (Wang et al., 2022). Such clonotype clusters are generally restricted to BCRs with the same CDR3 length (Satpathy et al., 2015); Since many antibodies whose CDR3 length differs by 1-2 amino acids have been found to target the same epitope (D'Angelo et al., 2018; Wong et al., 2019), there is benefit in adding flexibility to BCR clustering. Moreover, antibodies with different V and J genes or with CDR3 sequence identities below 80% have been found to target the same anti-SARS-CoV-2 RBD epitopes (Barnes et al., 2020; Dejnirattisai et al., 2021) or NTD epitopes (Liu et al., 2021). In the case of the human antibody repertoires, overlap between donors as defined by clonotyping antibody sequences is about 0.3% among three healthy adult donors and 0.1% among three cord blood samples (Soto et al., 2019). In order to increase sensitivity, our group constructed a clustering tool on the InterClone web server that provides a more flexible thresholds CDR similarity (Wilamowski et al., 2022). This method assumes that antibodies within a CDR sequence identity are more likely to target the same epitope. A detailed explanation of this method and the process of a realistic application are described below.

Recently, two groups discovered a set of 11 SARS-CoV-2 infection enhancing antibodies (Li et al., 2021; Liu et al., 2021). We sought to identify such infection-enhancing antibodies in a large-scale antibody repertoire sequence data from COVID-19 patients and healthy donors (Ismanto et al., 2022). Because enhancing antibodies bind their antigen primarily *via* their heavy chain, as captured in the Cryo-EM structure data (PDB ID 7LAB, 7DZX, 7DZY), we focused on heavy chain CDRs. Moreover, since we did not know in advance the safest sequence identity threshold to use for each CDR, we used 80% for CDRH1 and CDRH2 and 60% for CDRH3. Although we could find antigen binders within these thresholds, the false positive rate was quite high (more than 80%) (Ismanto et al., 2022). A safer threshold seems to be 90% for CDRH1 and CDRH2 and 70% for CDRH3. Donor antigen exposure was a critical factor in the false positive rate. We found that the true enhancing antibody rate was approximately 100 times higher in COVID-19 patients than in healthy donors. Unlike other web servers (e.g., Vidjil, AbYsis, OAS, etc.) (Duez et al., 2016; Swindells et al., 2017; Olsen et al., 2022), InterClone hosts a large database of BCR and TCR sequences and allows such flexible search or clustering operations on the stored data. InterClone also allows users to control data visibility.

## 6 Future perspectives

Human BCR repertoires are shaped by antigen exposure. A wide range of diseases, from infection, cancer, and autoimmunity, can shape our repertoires. Aging also has a profound effect on BCR (and TCR) diversity. For these reasons, BCR and TCR repertoires have attracted much attention as potential biomarkers for health, disease, vaccination, or other therapeutic activity. In recent months, two groups have reported the ability to clearly separate COVID-19 patients based on BCR repertoires (Ortega et al., 2021; Chen et al., 2022), and our own, unpublished findings support this ability. Therefore, it is reasonable to anticipate a new generation of biomarkers based on BCR repertoires that will drive forward technology in the four main areas discussed here (sorting, sequencing, analysis, and validation).

One of the distinguishing features of BCR-based biomarkers from conventional biomarkers is that the antibodies encoded by the BCR sequences are directly involved in the prevention or (as in the case of autoimmunity) mediation of disease. This implies that the downstream application of BCR-based biomarkers is a new generation of therapeutics that closely resemble our body’s own defence mechanisms. Computational methods, in particular the ability to accurately predict antibody-antigen interactions from repertoire data, will make a critical difference in these efforts. Given the recent breakthroughs in Deep learning, there is thus much cause for optimism in the coming years for repertoire-based biomarkers and therapeutics.

One topic we have not covered is antibody delivery. Antibodies are traditionally administered directly as proteins. Moreover, mRNA vaccination has proved to be a robust and safe way to induce neutralizing antibodies against COVID-19 (Chaudhary et al., 2021). mRNA vaccinations are now being designed against various antigen targets including Zika, influenza virus, CMV, Respiratory syncytial virus, Ebola, and HIV. At present, most mRNA vaccines encode one or more antigens (Barbier et al., 2022). Two studies have explored the feasibility of expressing antibodies against Respiratory syncytial virus and HIV *via* mRNA vaccination (Tiwari et al., 2018; Lindsay et al., 2020). In one study, researchers expressed whole palivizumab (neutralizing antibody of RSV) in the lung *via* synthetic mRNA delivery by intratracheal aerosol (Tiwari et al., 2018). Cells co-transfected with mRNAs encoding the light and heavy chains at a 1:4 molar ratio could efficiently form whole IgG antibodies and prevent detectable infection (Tiwari et al., 2018). In another study, mRNA that encoded a HIV neutralizing antibody (PGT121) as well as a membrane anchor protein, was used to efficiently localize the antibody to the cell surface and capture simian-HIV (Lindsay et al., 2020). Thus, it will be interesting to see whether antibody delivery can be routinely implemented as RNA.

Of the four technologies reviewed here (B cell sorting, BCR sequencing, BCR repertoire analysis, and Experimental validation), repertoire analysis is the one area that expected to become radically transformed by advances in computational science. While we can cluster antibodies into specificity groups using CDR or gene usage similarity, the sensitivity of such methods is severely limited. Such limitation, in turn, is due to poor coverage of well-annotated BCR sequences, as discussed in a research (Jespersen et al., 2019). Only a few antigens have been well studied, so machine learning models are currently unable to learn the patterns associated with specific epitopes. Therefore, the transformation from clustering based on similarity to the ability to predict epitopes will require steady progress in sorting, sequencing, and validation. Such progress will come through investment in repertoire-based analysis of various diseases, data sharing and basic infrastructure. The establishment of data standards is one important step. It remains uncertain whether data providers will merge together under government-sponsored institutions (e.g., NIH, EBI, AMED) or remain independently operated. In either scenario, it will be interesting to see how the pharmaceutical industry responds to the growing information contained in human BCR repertoires.
